# AcrVIA6 Is a Monomeric DNA‐Binding Protein That Does Not Directly Inhibit Cas13a

**DOI:** 10.1096/fj.202500684R

**Published:** 2025-06-20

**Authors:** Ju Hee Han, So Yeon Lee, Hyun Ho Park

**Affiliations:** ^1^ College of Pharmacy, Chung‐Ang University Seoul Republic of Korea; ^2^ Department of Global Innovative Drugs Graduate School of Chung‐Ang University Seoul Republic of Korea

**Keywords:** AcrVIA6, adaptive immunity, anti‐CRISPR, CRISPR‐Cas system, crystal structure

## Abstract

The CRISPR‐Cas system is a crucial adaptive immune mechanism in prokaryotes, providing defense against invading genetic elements. Among various CRISPR‐Cas systems, the type VI‐A system, employing RNA‐guided RNase Cas13a, has been extensively studied for its ability to target and degrade single‐stranded RNA. Anti‐CRISPR (Acr) proteins have evolved as natural inhibitors of these systems, with AcrVIA proteins specifically targeting the Cas13a enzyme. However, there is currently conflicting debate regarding the anti‐CRISPR function of AcrVIA6. This study reveals that AcrVIA6 functions as a DNA‐binding protein rather than a Cas13a inhibitor, as it does not block RNA cleavage. These findings challenge its role in CRISPR‐Cas regulation.

## Graphical abstract

AcrVIA6 was initially identified as an anti‐CRISPR (Acr) protein that inhibits Cas13a function, but its classification as a true Acr has been questioned in recent reports. Our findings support this controversy, showing that AcrVIA6 binds to double‐stranded DNA as a monomer and does not directly inhibit the enzymatic activity of Cas13a.

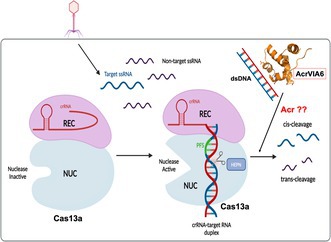

## 1 Introduction

The CRISPR‐Cas ( **C** lustered **R** egularly **I** nterspaced **S** hort **P** alindromic **R** epeats and **C** RISPR‐ **AS** sociated proteins) system is an adaptive immune mechanism in prokaryotes, conferring protection against invading genetic elements such as bacteriophages and plasmids [1, 2]. The system creates a genetic memory that guides Cas proteins to recognize and cleave complementary sequences during invasion by incorporating foreign DNA sequences, known as spacers, into the CRISPR array, thereby neutralizing threats [3]. The type VI‐A CRISPR‐Cas system, which utilizes RNA‐guided RNase Cas13a, has gained significant attention due to its ability to target and degrade single‐stranded RNA [4]. Owing to its versatility, this system has been used in various biotechnological applications, including RNA editing, viral detection, and gene silencing [5].

Anti‐CRISPR (Acr) proteins, which are naturally occurring inhibitors of CRISPR‐Cas systems, have evolved as a counterdefense mechanism employed by bacteriophages and other mobile genetic elements [6]. These proteins exhibit diverse inhibitory mechanisms, targeting different stages of the CRISPR‐Cas immune response from blocking spacer acquisition to inhibiting interference activities. The AcrVIA6 protein, which targets the type VI‐A CRISPR‐Cas system, has been identified as a potent Cas13a inhibitor [4]. However, the function of AcrIVA6 has become the subject of controversy. While an initial study suggested that AcrIVA6 is a true anti‐CRISPR protein, another study argued that AcrIVA6 might not be an anti‐CRISPR due to its inability to inhibit Cas13 activity in an in vivo system [7].

Therefore, this study presents structural and biochemical evidence demonstrating that AcrIVA6 functions as a DNA‐binding protein rather than a Cas13a inhibitor. Our findings reveal that AcrIVA6 binds to double‐stranded DNA (dsDNA) as a monomer and does not inhibit RNA cleavage by Cas13a in vitro. These results challenge the current understanding of AcrIVA6 as a functional Acr protein, providing new insights into its role and further contributing to the ongoing debate within the field.

## 2 Results

**FIGURE 1:**
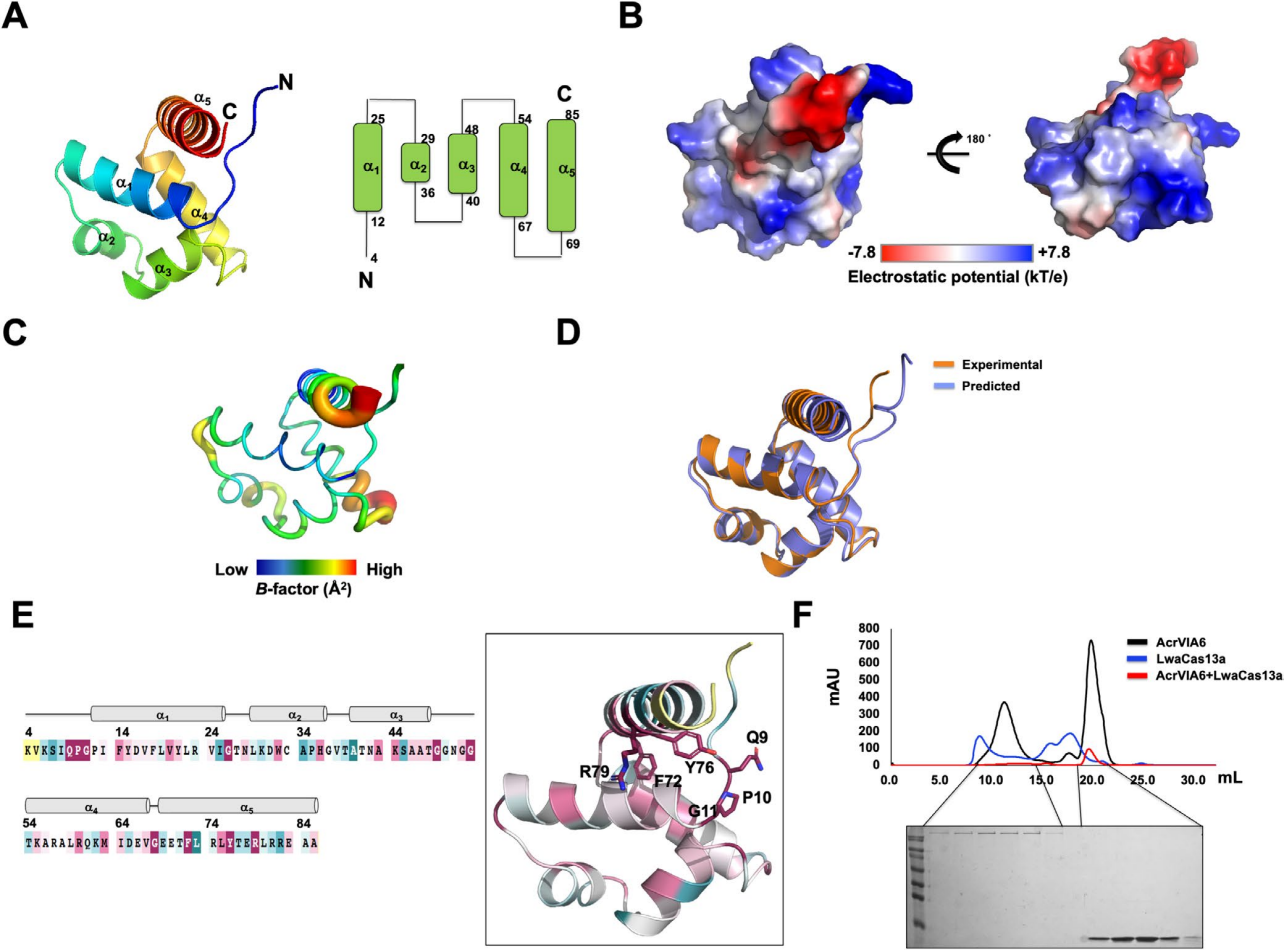
The overall structure of AcrVIA6. (A) A ribbon diagram of monomeric AcrVIA6 that is illustrated with a rainbow color gradient, progressing from the N‐terminus to the C‐terminus. The topology diagram of the AcrVIA6 structure is provided at the right panel. (B) The electrostatic surface representation of AcrVIA6, with the scale ranging from −7.8 kT/e (red) to + 7.8 kT/e (blue). (C) A putty model illustrates the distribution of B‐factors, using a rainbow gradient from red to violet to indicate the varying B‐factor values. (D) Comparison of the experimental crystal structure of AcrVIA6 with the predicted structure generated by AlphaFold2. (E) The amino acid sequence of AcrVIA6 is displayed with colors representing the level of sequence conservation based on ConSurf analysis. The cartoon representation of AcrVIA6, where colors reflect the degree of amino acid sequence conservation from ConSurf analysis, with the most conserved residues labeled. (F) Analysis of the interaction between AcrVIA6 and Cas13a by SDS‐PAGE following SEC. SDS‐PAGE gels produced by loading the SEC fractions indicated by the black lines are shown below the SEC profiles.

We purified AcrVIA6 using affinity and size‐exclusion chromatography (SEC) to study its Cas13a inhibition mechanism and functional controversy. The protein was crystallized, and its structure was solved at 2.39 Å. The high‐resolution structure of AcrVIA6 revealed a five‐helical bundle fold, modeled from residues 4–85 (Figure 1A). The helices (α1–α5) span residues 12–25, 29–36, 40–48, 54–67, and 69–85 (Figure 1A). Electrostatic analysis showed predominantly positive charges (Figure 1B). *B*‐factor analysis indicated overall rigidity (avg. 28.5 Å), except for the α4 start and C‐terminal region (avg. 48.2 Å) (Figure 1C). Superposition with the Alphafold2‐predicted model showed overall similarity, but with significant N‐terminal loop differences (Figure 1D). Finally, we analyzed the amino acid conservation using the Consurf server to infer functionally important residues in AcrVIA6 [8]. This analysis revealed that Q9, P10, and G11, belonging to the N‐terminal loop, as well as F72, Y76, and R79 in the last helix α5, are completely conserved, along with several conserved glycine residues in the middle (Figure 1E). Therefore, those conserved amino acids likely play a crucial role in the function of AcrVIA6.

After analyzing the AcrVIA6 structure, we determined how AcrVIA6 inhibits Cas13a function to investigate whether AcrVIA6 directly interacts with Cas13a. Since many Acr proteins inhibit the function of their target nucleases by directly binding to them [9], we tested this direct interaction first. Accordingly, we purified both AcrVIA6 and Cas13a proteins and mixed them, analyzing their binding through size‐exclusion chromatography (SEC). SEC followed by SDS‐PAGE showed that the two proteins did not coexist and comigrate on the SDS‐PAGE (Figure 1F). Hence, AcrVIA6 does not bind to Cas13a from *Leptotrichia wadei* (LwaCas13a).

**FIGURE 2:**
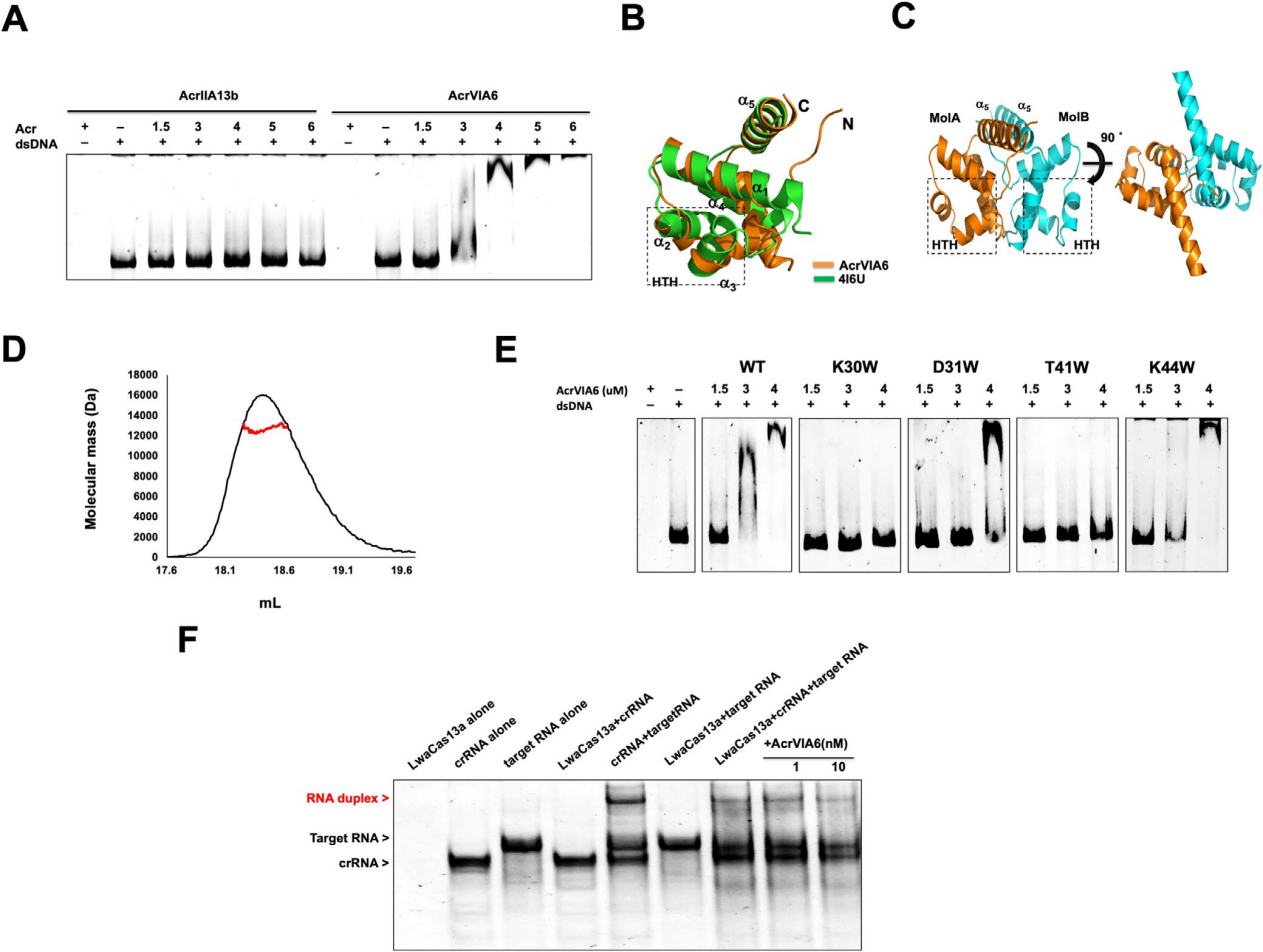
AcrVIA6 is a monomeric DNA‐binding protein that cannot directly inhibit Cas13a. (A) Representative EMSA with AcrVIA6 using dsDNA as a substrate. Purified AcrVIA6 at the indicated concentrations was mixed with substrate DNA. Nondenaturing acrylamide gels stained with SYBR Gold are shown. AcrIIA13b, which is another family of similarly sized Acr that cannot bind dsDNA, was used as a negative control. + and – indicate added and not added, respectively. (B) Structural comparison of AcrVIA6 with transcriptional regulator C.Esp1386I (PDBid: 4I6U), which was picked as the most structurally similar regulator to AcrVIA6 by DALI server via superimposition. (C) The model of AcrVIA6 dimeric structure was generated based on the dimeric structure of transcriptional regulator C.Esp1386I. Putative HTH motif that might be critical for dsDNA binding, and α5 helix that might be important for dimer formation. (D) The MALS profile from the SEC peak, with experimental data presented as a red line. The X‐axis denotes the SEC elution volume, while the Y‐axis represents the distributions of the absolute molecular mass. (E) EMSA with the indicated AcrVIA6 mutants using dsDNA in various protein concentrations. Nondenaturing acrylamide gels stained with SYBR Gold are shown. + and – indicate added and not added, respectively. (F) An in vitro anti‐CRISPR activity assay was used to detect a concentration‐dependent inhibition of LwaCas13a activity by Acr. Polyacrylamide gels (4%) were stained with SYBR Gold. The amount of AcrVIA6 added to the reaction is indicated.

To explore AcrVIA6 function, we used the DALI server to identify structurally similar, functionally characterized proteins. The top five hits, including the transcription regulator C.Esp1396I, were all DNA‐binding proteins. Given these findings, we tested AcrVIA6's DNA‐binding ability via EMSA, which confirmed concentration‐dependent binding to dsDNA. In contrast, AcrIIA13b, a non‐DNA‐binding anti‐CRISPR protein of similar size, showed no binding, confirming AcrVIA6 as a DNA‐binding protein (Figure 2A).

Then, we compared the AcrVIA6 structure with that of transcription regulator C.Esp 1396I (PDB id: 4I6U; hereafter called 4I6U), which is structurally most similar to AcrVIA6 with a well‐studied DNA‐binding mechanism, to analyze how AcrVIA6 binds to dsDNA [10]. Structural superposition revealed that 4I6U comprises five helices and shows significant structural similarity to AcrVIA6 (RMSD: 2.7 Å) (Figure 2B). Notably, the helix‐turn‐helix (HTH) motif in 4I6U, formed by α2 and α3 helices and directly involved in dsDNA binding, is well conserved in AcrVIA6 (Figure 2B). Thus, AcrVIA6 may also utilize the HTH motif, similar to 4I6U, to bind dsDNA. We modeled the dimer structure of AcrVIA6 based on the functional 4I6U dimer structure since 4I6U and most other structurally similar proteins, including CLGR (PDB id: 3F51) [11] and MUTR (PDB id: 6W1F) [12], bind DNA as dimers, with the formation of the dimer being significantly influenced by α5 (Figure 2C). The resulting AcrVIA6 dimer structure was very similar to those of other structurally similar proteins (Figure 2C). Afterward, we analyzed AcrVIA6 stoichiometry in solution using the SEC‐MALS to determine whether it functions as a dimer. The experimentally determined molecular weight of AcrVIA6 was 11.2 kDa, whereas its theoretical molecular weight is 10.5 kDa (Figure 2D). Hence, AcrVIA6 exists as a monomer in a solution, unlike other structurally similar DNA‐binding proteins that form dimers. After confirming that AcrVIA6 exists as a monomer, we revisited the modeled dimer structure in more detail. We found that the N‐terminal loop of AcrVIA6 interferes with dimer formation due to steric hindrance with α5, which plays a crucial role in dimerization, while the overall dimer construction of AcrVIA6 is similar to that of other structurally similar proteins. The long N‐terminal loop, impeding dimer formation, is a distinctive feature of AcrVIA6 and contributes to its binding to dsDNA as a monomer.

Finally, we identified amino acids in the putative HTH motif that might be crucial for DNA binding, created mutations in these residues, and analyzed how these mutants influence DNA binding to verify the accuracy of our proposed model of DNA binding by AcrVIA6. Specifically, we examined homologous proteins of AcrVIA6 to identify conserved amino acids, particularly those in the HTH motif, and identified surface‐exposed amino acids among these residues that are probably involved in the functional activity of this protein. Thus, we found that residues K30, D31, T41, N42, and K44 are likely important for DNA binding. Consequently, we substituted these residues with tryptophan (W) to disrupt DNA binding. Then, we conducted the EMSA again to analyze the effects of these mutants. As predicted, K30W and T41W mutants completely lost their DNA binding ability, while D31W and K44W mutants showed weakened binding (Figure 2E). Therefore, AcrVIA6 is a dsDNA‐binding protein that uses the HTH motif, comprising α2 and α3, to bind dsDNA.

## 3 Discussion

Cas13a, an RNA‐targeting enzyme in bacterial and archaeal CRISPR‐Cas systems, degrades RNA unlike Cas9, which cleaves DNA. Its programmable RNA‐targeting ability enables applications in RNA interference, transcriptome engineering, and antivirals. Acr proteins inhibit CRISPR‐Cas via diverse mechanisms, with AcrVIA1–AcrVIA7 recently identified as Cas13a inhibitors.

Cas13a is an RNA‐targeting enzyme that belongs to the CRISPR‐Cas family of adaptive immune systems in bacteria and archaea. Unlike the more well‐known Cas9, which cleaves double‐stranded DNA, Cas13a recognizes and degrades RNA molecules. Acr proteins inhibit CRISPR‐Cas systems through various mechanisms. A group of Cas13a inhibitors ranging from AcrVIA1 to AcrVIA7 has been predicted recently [13]. However, the anti‐CRISPR function of these proteins remains unclear. Recent studies have suggested that the identification of the AcrVIA family may have been based on inaccurate bioinformatic analyses, and assays, such as plaque assays, have shown no inhibitory activity [7]. Therefore, the true function of these proteins as the AcrVIA family is still unclear.

This study demonstrated through structural and biochemical analyses that AcrVIA6 is a DNA‐binding protein with the HTH motif and functions as a monomer. Additionally, we found that AcrVIA6 does not directly interact with Cas13a. Since AcrVIA6 binds DNA, its function may be unrelated to Cas13a, considering that Cas13a is an RNA‐targeting enzyme that cleaves target RNA in conjunction with crRNA. Therefore, DNA binding alone would not directly inhibit Cas13a. Using an in vitro Cas13a activity assay, we confirmed that AcrVIA6 fails to inhibit RNA cleavage, even at high concentrations (Figure 2F). Hence, along with our structural and biochemical studies, AcrVIA6 seems to function as a DNA‐binding protein rather than as a Cas13a inhibitor. This idea is further supported by the fact that some proteins within Acr operons, such as Aca proteins, regulate the expression of Acrs or Cas proteins for CRISPR‐Cas systems by acting as transcriptional regulators [14]. Thus, AcrVIA6 may serve the role of a transcription regulator.

Although our study has certain limitations, such as the absence of a positive control for a known Cas13a inhibitor and testing with only one bacterial Cas13a, all our data suggest that AcrVIA6 is unlikely to function as an anti‐CRISPR protein. Instead, it may play a regulatory role, potentially controlling transcription related to the CRISPR‐Cas system. The significance and functional role of AcrVIA6's DNA binding ability should be elucidated in future research.

## Author Contributions

H.H.P. designed and supervised the project. J.H.H. performed all the experiments, including collecting biochemical and structural data. J.H.H. and S.Y.L. solved the structure. H.H.P. and J.H.H. wrote the manuscript. All the authors discussed the results and commented on the manuscript.

## Conflicts of Interest

The authors declare no conflicts of interest.

## Data Availability

Stored in repository.
